# NF-κB, a culprit of both inflamm-ageing and declining immunity?

**DOI:** 10.1186/s12979-022-00277-w

**Published:** 2022-05-17

**Authors:** Preeyaporn Songkiatisak, Shah Md Toufiqur Rahman, Mohammad Aqdas, Myong-Hee Sung

**Affiliations:** Laboratory of Molecular Biology and Immunology, National Institute on Aging, National Institutes of Health, 21224 Baltimore, MD USA

**Keywords:** NF-κB, Inflammation, Ageing, Immunosenescence, Mammalian stress pathways

## Abstract

NF-κB is generally recognized as an important regulator of ageing, through its roles in cellular senescence and inflammatory pathways. Activated in virtually all cell-cell communication networks of the immune system, NF-κB is thought to affect age-related defects of both innate and adaptive immune cells, relevant to inflamm-ageing and declining adaptive immunity, respectively. Moreover, the family of NF-κB proteins that exist as heterodimers and homodimers exert their function beyond the immune system. Given their involvement in diverse areas such as DNA damage to metabolism, NF-κB has the potential to serve as linkages between known hallmarks of ageing. However, the complexity of NF-κB dimer composition, dynamic signaling, and tissue-specific actions has received relatively little attention in ageing research. Here, we discuss some areas where further research may bear fruit in our understanding the impact of NF-κB in healthy ageing and longevity.

## Background

Ageing is one of the few universal features that directly impact all animals, including humans. Even though age-associated diseases or degeneration that limit lifespan may differ between individuals, there are recurring themes in the biological process of organismal ageing that had been termed the “hallmarks of ageing” [1]. While the existing concepts have fueled intense examination of several pathways such as nutrient sensing mTOR, SIRT1, p53, IGF-1, and telomere attrition, it is equally important to identify connections between seemingly unrelated mechanisms of ageing, or to discover new aspects of ageing biology. One of the master transcriptional regulators at the crossroads of immunity and ageing is nuclear factor kappa B (NF-κB), with its diverse roles implicated in nearly all the hallmarks of ageing.

**Fig. 1:**
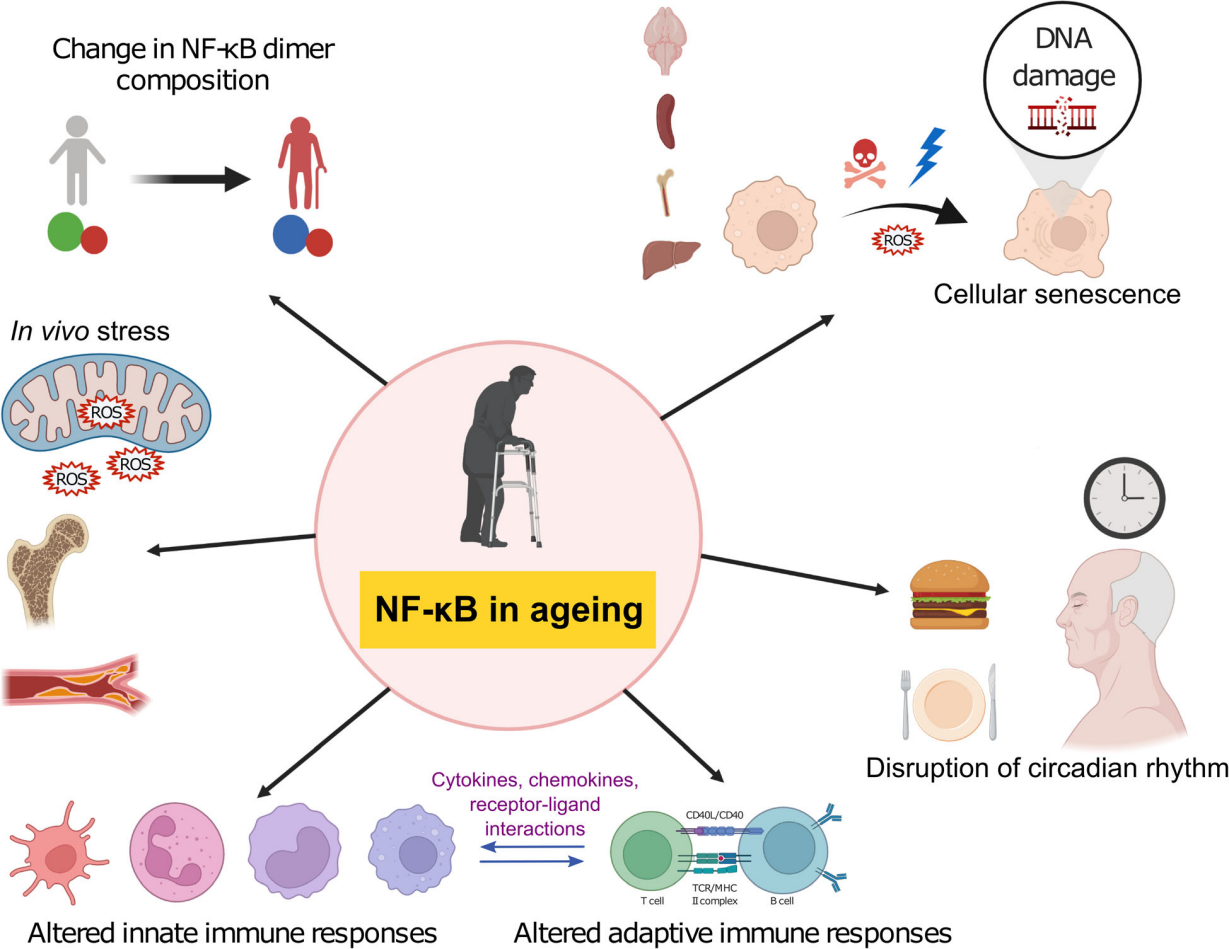
NF-κB and mechanisms of ageing. Various aspects of ageing that activate NF-κB signaling, or ageing mechanisms which are regulated by NF-κB

In this mini review, we aim to expose relatively unexplored topics surrounding NF-κB in the spirit of ‘leave no stone unturned’ (Fig. 1). In both the innate and the adaptive immune systems, NF-κB senses danger signals and regulates the expression of cytokines and their receptors in a complex cell-cell communication cascade. Therefore, any age-related intrinsic defects of NF-κB signaling would have a direct impact on cell-cell communications within the immune system and with the surrounding microenvironments. Here we discuss evidence and ideas for the relevance of NF-κB in two concepts of immune ageing: inflamm-ageing and declining adaptive immunity (immuno-senescence). The cited studies are not necessarily drawn from the ageing research community. Rather, we attempted to compile independent reports which, when connected in the context of ageing, suggest potential age-related pathophysiological mechanisms. Thus, emphasis is on the areas that may warrant further investigations in the future, based on what has been learned so far.

### Growing old alongside the complex structure and signaling of NF-κB

NF-κB is a ubiquitously expressed transcription factor which regulates expression of genes responsible for various biological processes, including immune responses, inflammation, cell proliferation, and apoptosis [2]. In mammalian cells, NF-κB exists as homodimers or heterodimers consisting of five members of the Rel family proteins which contain the DNA binding Rel homology domain (RHD), namely RelA (also known as p65), RelB, c-Rel, NF-κB1/p50, and NF-κB2/p52. The transcriptionally inactive p50 and p52 are generated from the precursors p105 and p100, respectively. These NF-κB subunits combine with each other to form different dimer molecules [3]. In unstimulated cells, the NF-κB dimers bind to the inhibitor of NF-κB proteins (IκBs), which are sequestered in the cytoplasm. Upon stimulation with various extracellular stimuli or stress, IκBs get degraded, the free NF-κB dimers translocate into the nucleus and reversibly bind to specific DNA binding motifs, and modulate the expression of hundreds of target genes [4]. As each of the dimer molecule has different affinities for consensus DNA binding motifs [5], a distinct gene expression profile might be induced depending on which dimers translocate into the nucleus and bind to the regulatory sites in the genome. Any changes in the NF-κB dimer composition would affect the DNA binding profile and consequent gene expression programs. Using the steady-state fluorescence anisotropy assay, Ramsey et al. have shown that the differential presence of IκBs alters the equilibrium concentrations of NF-κB dimers [6]. Thus, any changes in the expression of NF-κB subunits or the inhibitory IκB proteins would affect the NF-κB:DNA interactions and gene expression as a consequence.

Although alterations of the NF-κB pathway have been reported in physiological ageing, no study has been carried out to directly determine the changes in NF-κB dimer composition with ageing. Genetic perturbation studies have used knock-out or overexpression of one or more subunits of NF-κB or IκBs which alter the equilibrium dimer composition. These studies indirectly suggest that altered NF-κB dimer composition may be an important factor in ageing. *Nfκb1*^*−/−*^ mice deficient in both the p105 precursor and p50 subunit show constitutive activation of NF-κB by RelA/RelA homodimer, which promotes premature ageing phenotype [7, 8]. Deletion of c-Rel subunit in mouse model develops Parkinson’s diseases (PD)-like neuropathology with ageing [9, 10]. On the other hand, systematic deletion of IκBα from the CNS in mice induces activation of NF-κB in neurons and astroglia and leads to increased Aβ production and promotes Alzheimer´s disease (AD) progression at an early age [11]. In *Sirt6-/-* mice, which have an accelerated ageing phenotype, the haploinsufficiency of RelA results in improved growth and a longer lifespan in *Sirt6-/-Rela+/-* mice compare to the control *Sirt6-/-* littermates [12]. The RelA haploinsufficiency in aged muscle-derived stem/progenitor cells (MDSPCs) led to increased myogenic potential and higher resistance to oxidative stress [13].

Direct measurements of the NF-κB dimer composition in physiological ageing require a longitudinal study of animal models using either a sensitive and high-throughput single-cell proteomics assay or live-cell analyses of dimers in individual cells. Savas Tay’s group has proposed a single-cell multiplex analysis method termed proximity-sequencing (Prox-seq) to simultaneously measure proteins and protein complexes as well as mRNAs in individual cells. This newly developed method can be leveraged for the quantitative measurement of NF-κB dimer composition in single cells and the changes with ageing [14]. Using the number and brightness assay, Martin et al. [15] have demonstrated a quantitative measurement technique for the live-cell measurement of NF-κB dimer composition, reporting that the proportion of RelA:RelA homodimers is higher than expected in the nucleus of TNF-α stimulated fibroblast cells. A few groups [16, 17] have generated fluorescent knock-in reporter mouse models expressing a fluorescent fusion of the RelA subunit from the endogenous locus. We have also generated mouse models expressing RelA and/or c-Rel subunit fused to either mEGFP and/or mScarlet. These mouse models can be used as a tool to measure the endogenous NF-κB dimer composition using fluorescent correlation or cross-correlation spectroscopy (FCS or FCCS) assays in live cells. These experimental techniques will help answer the question how NF-κB dimer composition changes with ageing.

Apart from the NF-κB dimer composition, post-transcriptional modifications such as the site-specific phosphorylation of the NF-κB subunits can affect the interactions, stability, degradation, and transcriptional activity of NF-κB dimers. Certain phosphorylation events might control the selectivity of NF-κB transcriptional activity in a gene-specific manner. The phosphorylation status of all the NF-κB subunits, the corresponding activating protein kinases, and their potential biological effects have been reviewed elsewhere [18]. However, only a handful of studies have reported age-associated changes in phosphorylation status of different NF-κB subunits. During skin ageing, increased phosphorylation of IKKα (Thr23) was observed in the nucleus which induced the phosphorylation of RelA at Ser536 and enhanced the NF-κB activity through increased DNA binding [19]. Another study reported an increase of phosphorylated RelA in hypothalamus and cortical tissues with ageing [20]. More longitudinal studies are required to understand the functional relevance of all the possible age-related changes in post-translational modifications of NF-κB subunits in ageing biology.

### From stress to signaling: hypertension, mechanical tissue strain, and oxidative stress can activate NF-κB

There are various *in vivo* stresses that can activate the NF-κB signaling pathway including shear stress, mechanical strain, and oxidative stress. Generally, arterial endothelial cells experience mainly two types of shear stresses: pulsatile shear stress (PS) and oscillatory shear stresses (OS). In the human umbilical vein endothelial cells (HUVEC), NF-κB appeared to be activated in response to PS and OS flows [21]. During the development of atherosclerosis, shear stress in endothelial cells (ECs) and smooth muscle alters NF-κB signaling which induces pro-inflammation cytokines, chemokines, and adhesion molecules in vascular ECs, promoting monocyte recruitment and disease progression [22, 23]. In addition, acute shear stress in ECs appears to activate NF-κB in an integrin-dependent manner. The signaling downstream of integrin triggers activation of the classical NF-κB pathway [23].

Age-related diseases including osteoporosis and sarcopenia may be conditions where NF-κB is activated via mechanical strains. Exposure of articular cartilage to excessive mechanical loading is strongly associated with the pathogenesis of osteoarthritis. Gremlin-1 is a mechanical loading-inducible factor in chondrocytes and activates NF-κB signaling, resulting in a subsequent induction of catabolic enzymes [24]. Sarcopenia, defined as the loss of skeletal muscle mass and strength, is a common feature of ageing. Muscle unloading or loss of muscle innervation led to an 8-fold increase in NF-κB activation [25]. Studies also illustrate a constitutive activation of NF-κB in aged muscle, even though the mechanism in the context of sarcopenia is still unclear [26]. In addition, mechanical stimulation could activate the classical NF-κB pathway in osteoblasts and related cells [27]. Depending on the degree of the stimulus, the NF-κB pathway is either inhibited by low tensile changes or activated by high stresses [27].

NF-κB is recognized as a redox-sensitive transcription factor and involved in the cellular response to oxidative stress [28]. With ageing, oxidative stress is accumulated due to higher rates of reactive oxidative species (ROS) generation, mitochondrial dysfunction, defects in electron transport, and additional oxidative stress from other age-related conditions [28, 29]. Exposure to ROS causes damages to macromolecules, including DNA, proteins, and lipids. Interestingly, ROS are important regulator of TNF-α signaling which can promote either NF-κB activation or inhibition leading to cell survival or death [30]. NF-κB heterodimers may be modified when cells accumulate oxidative stress. For example, Cys-62 in the RHD domain of p50 subunit may be oxidized, potentially leading to increased DNA binding [31]. Phosphorylation of Ser-276 on RelA has been shown to be ROS-dependent, which is required for transcriptional activation of some NF-κB target genes [31].

### Tissue-specific senescence and SASP of macrophages and their vulnerability to DNA damage in ageing

Cellular senescence is a state of stable cell cycle arrest, generally considered to fuel the ageing process [32]. Senescent cells secrete various pro-inflammatory cytokines, chemokines, growth and angiogenic factors, referred to as senescence-associated secretory phenotype (SASP) [33, 34]. SASP recruits other immune cells via chemotaxis, one such cell type being macrophages. Macrophages are professional phagocytic cells that play a key role in the physiological clearance of dying cells and senescent cells [35, 36]. But the other side of the story is that macrophages themselves may become senescent in age-related inflammation. Expression of p16^INK4a^ and senescence-associated beta-galactosidase (SA-β-Gal) in macrophages increase with age and might amplify the cellular senescence and the SASP during ageing [37–40]. There is a two-way interaction between the senescent cells and macrophages, known as paracrine senescence [38], where either of the cells can be the first to show increased p16^INK4a^ expression. Also, senescent cells can skew macrophage phenotypes in a context-dependent manner. p53-expressing senescent stellate cells induced M1 polarization of macrophages [41], whereas senescent thyrocytes induced M2 polarization in macrophages [42]. The microenvironment within the ageing tissue can fuel the senescence in tissue-resident macrophages such as microglia [43], Kupffer cells [44], splenic macrophages [45], alveolar macrophages [46] and peritoneal macrophages [47]. Increased cell volume, shortened life-span, and disordered distribution of tissue-resident macrophages with age may reduce their interaction with surrounding cells and hamper their function [48]. However, the *in vivo* effects of senescent tissue-resident macrophages need to be investigated further.

Another factor that triggers senescence is accumulated DNA damage. Various pharmacological agents (chemotherapeutics), genotoxic agents (ionizing UV radiation), and oxidative stress are known to induce senescence. Senescent cells carry persistent nuclear DNA damage foci called as DNA-SCARSs (DNA segments with chromatin alterations reinforcing senescence). DNA-SCARSs lack the DNA repair proteins RPA and RAD51 and associate with promyelocytic leukemia (PML) nuclear bodies. They also harbor activated CHK2 and p53 molecules [49]. Protein damage or proteotoxicity induced by molecules such as ROS, protein tyrosine phosphatases, and Lipofuscin can also influence the senescence process [50, 51]. Senescent cells are also reported to show altered lipid metabolism, such as ROS-induced lipid damage, lipid deposition, and lipid modifications [52, 53].

The ataxia telangiectasia mutated (ATM) kinase is thought to play a critical role in the initiation of SASP through the DNA damage response [54, 55]. ATM activation triggers several downstream pathways including NEMO, an NF-κB essential modulator. NEMO is phosphorylated at Ser85 by activated ATM, resulting in sumoylation and mono-ubiquitination of NEMO at Lys277 and 309. After post-translational modifications, the ATM/NEMO complexes are exported from the nucleus to the cytoplasm, activating the IKKα/β complex, leading to NF-κB signaling [55, 56].

Overall, cellular senescence has been recognized to promote ageing and an array of diseases. However, the heterogeneity and incomplete information about macrophage senescence in terms of cause and effects raise concerns in designing the drugs or chemotherapeutic agents that targets all senescent cells rather than a sub-population of maladaptive macrophages.

### Do NF-κB signaling dynamics get muffled as the language of cell-cell communication in ageing?

Cell-cell communications through direct interactions or messenger molecules are essential for the integrity and stress responses of multicellular organisms. Dysregulation of cell communication pathways is associated with ageing and thought to promote age-related diseases [57]. Although senescent cells can be found in almost all the tissues of aged animals and humans, the senescent populations might originate initially from a small number of damaged cells, and propagate through the neighboring and remote cells or tissues via SASP [57–59]. NF-κB acts as a major transcription factor on the chromatin of senescent cells, and thus controls both the cell-autonomous and non-cell-autonomous aspects of the senescence program [58]. The cell-to-cell communication and propagation of NF-κB signaling are also crucial for activation of innate immunity against bacterial infections. After infection with entero-invasive bacterium *Shigella flexneri* in Shigelosis, the host intestinal epithelial cells (IECs) show activation of NF-κB with IL-8 induction which propagates from the infected to uninfected adjacent cells and generates rapid amplification of IL-8 production by uninfected bystander cells [60]. Dysregulation of NF-κB signaling and consequent defects in balanced cellular communication thought to occur with ageing compromise the effectiveness of innate immune system function. Emerging evidence also suggest that infected immune cells can transfer the pathogen associated molecular patterns (PAMPs) and derived signaling molecules to the non-infected cells, which then activate the bystander cells to mount a self-sustaining and amplified innate immune response [61].

NF-κB signaling is widely studied in macrophages where they get activated by PAMPs and DAMPs and secrete a large array of cytokines and chemokines. NF-κB promotes the differentiation of macrophages into M1 and M2 phenotypes, and also regulates the differentiation of naive CD4 T cells into Th1 and Th17 subtypes [62, 63]. Both subunits RelA and c-Rel play crucial roles in mediating the TCR signaling in naïve T cells during their activation and differentiation into Th1, Th2, Th17, Tregs, and Tfh cells [64]. Collectively, both canonical and noncanonical NF-κB pathways are responsible for the generation and function of effectors of adaptive immunity. A key feature of bridging the innate and adaptive immunity, which is crucial for the immune system, is that antigen-presenting cells (APCs) prime and train the naïve T cells via MHC (Major Histocompatibility Class) I and II molecules. For example, the c-Rel subunit of NF-κB is important for the delicate interactions between T cells and APCs [65]. NF-κB is one of the key signaling pathways that support the successful interplay of different immune and non-immune cells for a functional host response acting on both inter-cellular and intra-cellular levels. Overall, altered features of NF-κB signaling dynamics, such as peak timing, amplitude, or duration, which may occur in ageing might have substantial effects on the above-mentioned cell-to-cell communications *in vivo*. It is possible that such quantitative changes, albeit subtle, may be sufficient for the emergence of senescence phenotypes and impairment of innate and adaptive immune responses [66, 67].

### Weakened tissue barriers may prime inflammatory NF-κB signaling

The physical barriers that prevent exchange of content between different tissue compartments are critically important for organismal health. Examples of such tissue boundaries include the blood-brain barrier (BBB), the gut epithelium, and the skin epidermis. The barrier function is particularly essential for tissues that are exposed to the environmental microbes and preventing them from reaching deeper tissues. However, the integrity of these tissue barriers is compromised in ageing [68, 69], which is thought to promote a low-grade chronic inflammatory state, termed inflamm-ageing.

The gut epithelium is exposed to the microbiota which undergoes age-associated compositional changes [70, 71]. Gut inflammation can be modulated by a variety of factors, including the longevity-associated factor SIRT1 that inhibits NF-κB inflammatory signaling [72]. Ageing or alcohol consumption may increase gut permeability and cause a leakage of microbial products, contributing to liver inflammation. However, the dose of alcohol that causes leaky gut is still debated [73]. Given that red wine consumption is generally considered beneficial due to the reported benefits of resveratrol for healthy ageing [74], there might be a trade-off between the effects of alcohol and resveratrol in wine. Notably, the doses of resveratrol that promote healthy ageing have not been determined [75–78].

The dose and timing of prior exposure to microbial products can skew subsequent responses with either tolerogenic or priming effects in macrophages [79]. While adaptive immune memory is generally advantageous for the host, “innate memory”, a more recently introduced concept, may be a double-edged sword [80]. Innate memory, albeit shorter-term than adaptive memory, may be constantly reinforced by elevated endotoxins leaking through the aged barriers, driving inflammaging at sites such as BBB, gut, and skin [68, 81].

### Circadian disruption and NF-κB in ageing

In populations with western-style diets, chronic inflamm-ageing processes may be accelerated by further inflammatory signaling and the alteration of circadian rhythm [82, 83]. In high fat diet (HFD)-induced obesity, the adipose tissue-resident macrophages (ATMs) play a pivotal role in modulation of pro-inflammatory cytokines such as TNF-α and IL-1β [84]. ATMs may be therapeutically targeted to ameliorate the obesogenic potential of HFD in ageing populations. Better understanding of how inflammatory signaling and circadian clock are altered in ATMs during HFD-induced obesity will be important for developing strategies that combine drugs, dietary interventions, and chronotherapy to counter age-related obesity.

The circadian clock system may also be involved in longevity-inducing mechanisms of calorie restriction (CR). In a recent study using accurate monitoring of animal behaviors, Joseph Takahashi and colleagues revealed an important caveat of CR studies. Calorie-restricted mice were binge-feeding on a narrow time window, confounding the interpretation of previous CR data for the role of food intake amount only [85]. Because time-restricted feeding within the metabolically active phase confers resistance to obesity [86], future studies should decouple the effects of CR versus circadian regulation in examination of diet effects on ageing.

Circadian rhythm and inflammatory signaling have generally been examined separately, and more studies need to uncover how the two pathways intersect with each other. Their crosstalk is gaining more attention after studies showed convincing evidence of direct interactions between transcriptional regulators of the two systems [87, 88]. Perhaps a most direct link is provided by the transcriptional activation of NF-κB by the Clock, a key regulator of the circadian clock [89]. Conversely, NF-κB was found to be required for maintaining circadian rhythms in mice [90].

## Conclusions

NF-κB is an essential transcription factor for regulating rapid innate responses and long-term adaptive immune responses and memory through T and B lymphocytes. Hence, it is uniquely positioned to affect the opposite aspects of the age-related immune dysregulation: inflamm-ageing (mediated largely by innate immune cells of the myeloid lineage) and declining adaptive immunity (through intrinsic defects of lymphocytes in signaling and proliferation). Building upon the exciting discoveries about the ageing of the immune system to date, the research community may find it rewarding to go beyond the “usual suspects”. For example, novel insights may come from studies of NF-κB and related mechanisms underlying the inter-relationships between different hallmarks of ageing. Resolving the relevant questions will require innovative technical approaches, improved animal models, as well as new conceptual frameworks.

## Data Availability

Not applicable.

## Abbreviations

AD: Alzheimer’s disease
ATM: Ataxia telangiectasia mutated
ATMs: Adipose tissue macrophages
BBB: Blood brain barrier
CNS: Central nervous system
CR: Calorie restriction
EC: Endothelial cell
DNA-SCARS: DNA segments with chromatin alterations reinforcing senescence
FCCS: Fluorescence cross-correlation spectroscopy
HFD: High fat diet
IEC: Intestinal epithelial cell
IL: Interleukin
mEGFP: Monomeric enhanced green fluorescent protein
NF-κB: Nuclear factor kappaB
PAMP: Pathogen-associated molecular pattern
RHD: Rel homology domain
ROS: Reactive oxygen species
SASP: Senescence-associated secretory phenotype
TCR: T cell receptor
Th: T helper cell
TNF: Tumor necrosis factor
UV: Ultraviolet
